# P09 Incidence of bacterascites and spontaneous bacterial peritonitis (SBP) at a tertiary care teaching hospital

**DOI:** 10.1093/jacamr/dlaf230.016

**Published:** 2025-12-04

## Abstract

**Background:**

Microbiology laboratory receives samples from day-case units for patients undergoing elective drainage of ascites for symptomatic relief only as well as inpatients with suspicion of SBP. The elective samples usually grow organisms of no clinical significance.

**Background:**

SBP is an infection of ascitic fluid in the absence of any intra-abdominal, surgically treatable source of infection. Also defined by ascitic neutrophil count >250 cells/mm^3^ or total ascitic white cell count >500 cells/mm^3^. Bacterascites may represent a transient and spontaneously reversible colonization of ascites but for those who are symptomatic, it may herald SBP. In bacterascites, the ascitic neutrophil count is <250 cells/mm^3^ but with a positive fluid culture.

**Objectives:**

To find the incidence of bacterascites and SBP in the ascitic fluid samples sent from the day-case unit, yield from blood culture bottles, pathogens detected and correlation to symptoms.

**Methods:**

All ascitic fluid samples sent from day unit and inpatients from January 2025 to March 2025 were reviewed using trust’s databases.

**Results:**

A total of 103 ascitic fluid samples were received. 94 samples were from inpatient and 09 from outpatient/day-case unit. Only 10 samples were culture positive—all from inpatient (9.7%). None of the elective samples from the day case unit had cell count more than 200 and all were culture negative. None of these patients had symptoms of SBP and they underwent drainage for symptomatic relief only. Only 15 samples from inpatient (15.9%) had cell count greater than 250 out of which only 3 were culture positive (*Staphylococcus epidermidis*, *Moraxella osloensis* and *Bacteroides fragilis*). Two of these patients had bowel obstruction and one was done for symptomatic relief. A total of 6 inpatient samples (6.4%) with WCC <250 were culture positive but grew organisms of no clinical significance (mainly ConS and *Bacillus subtilis* but these patients did not have any symptoms of SBP.

**Conclusions:**

We may be able to decrease the burden for laboratories by sending a sample for a cell count only from elective asymptomatic patients and performing a culture if the cell count is high.

